# Management of the Medico-Legal Dispute of Healthcare-Related SARS-CoV-2 Infections: Evaluation Criteria and Case Study in a Large University Hospital in Northwest Italy from 2020 to 2021

**DOI:** 10.3390/ijerph192416764

**Published:** 2022-12-14

**Authors:** Rosario Barranco, Isabella Caristo, Filippo Spigno, Marta Ponzano, Alessio Trevisan, Alessio Signori, Antonio Di Biagio, Francesco Ventura

**Affiliations:** 1 Department of Legal and Forensic Medicine, Health Science Department (DISSAL), University of Genova, 16132 Genova, Italy; 2 Department of Health Sciences, Section of Biostatistics, University of Genova, 16132 Genova, Italy; 3 Transfusion Medicine, Policlinico San Martino Hospital, 16132 Genova, Italy; 4 Infectious Diseases Unit, Policlinico San Martino Hospital, 16132 Genova, Italy; 5 Health Science Department (DISSAL), University of Genova, 16132 Genova, Italy; 6 IRCCS–Ospedale Policlinico San Martino Teaching Hospital, 16132 Genova, Italy

**Keywords:** SARS-CoV-2, healthcare-related SARS-CoV-2, medico-legal disputes, legal medicine, COVID-19

## Abstract

Healthcare-related SARS-CoV-2 infection is an issue of particular concern during the pandemic. It has important repercussions on the National Health System, which represents a source of medical-legal health disputes. In the healthcare context, there are reports of negative screening at hospital admission (via nasopharyngeal swabs) and subsequent diagnosis of SARS-CoV-2 infection during hospitalization. Such cases cannot be considered a priori of healthcare-related infections but require extensive in-depth evaluation. In this study, we propose an empirical classification to frame cases of SARS-CoV-2 infection diagnosed in the hospital (first negative admission swab, with subsequent positive test during hospitalization). The classification is based on five categories: nosocomial, probably nosocomial, indeterminate, probably community, and community cases. We analyzed patients who died after testing positive for SARS-CoV-2 during hospitalization (with initial negative screening) in the largest hospital in Northwest Italy from February 2020 to 31 December 2021. A total of 383 cases were tracked and are listed as follows: 41 cases (11%) were classified as nosocomial (i.e., 3.2% of COVID-19 deaths). In contrast, 71 cases (19%) were classified as probably nosocomial, 69 (18%) were indeterminate (i.e., the clinical, radiological, and laboratory characteristics did not provide information on the genesis of the infection), 166 (43%) were classified as probably community cases, and 36 (9%) were defined as community cases. Deceased patients with nosocomial SARS-CoV-2 infection constituted the following: 3.23% (41/1266) with respect to the total number of COVID-19 deaths, 1.1% (41/3789) with respect to those who entered the hospital with a negative swab and 0.82% (41/4672) with respect to the total of deaths from any cause of death. In this paper we discuss the topic and issues of nosocomial COVID-19 in hospitalized patients and address the medicolegal implications.

## 1. Introduction

Despite numerous infection-containment prevention measures, several cases of healthcare-related SARS-CoV-2 infection have been reported, starting in the first months of the pandemic [1]. The latter have important repercussions in terms of public health, risk management, control of healthcare-acquired infections (HAIs), and in the field of medical-legal disputes. Thus, nosocomial SARS-CoV-2 infections can be a source of compensation [1].

Since the first pandemic emergency phase, reports that addressed the problem of the spread of SARS-CoV-2 in a nosocomial environment with extremely heterogeneous incidence rates have been published [1].

According to the literature, an important share of hospital cases (about 20%) is nosocomial [2]. However, the results published in the literature are not always unambiguous: According to some studies, nosocomial COVID-19 is not associated with an increase in mortality [2,3], while further scientific research [4] suggests outcomes worst in cases of care-associated SARS-CoV-2. These results can be explained by various factors: On the one hand, nosocomial infection generally already affects patients with other comorbidities and therefore more fragile, on the other hand, the onset of a nosocomial context guarantees a more timely diagnosis and support. However, it is unclear whether different variants of SARS-CoV-2 lead to different outcomes. According to the literature, SARS-CoV-2 variants can potentially affect clinical outcomes [5].

During the first pandemic phase, the causes of SARS-CoV-2 infection related to assistance should be traced to insufficient isolation or to a lack or incorrect use of personal protective measures (PPE) [6]. In this sense, contagion in hospitals can essentially take place in the following ways: through indirect contact, contaminated surfaces or instruments (not adequately sterilized), or through close and unprotected contact (or inadequate use of PPE) with other patients and healthcare personnel.

However, in the literature [7] there is also an extreme heterogeneity in reference to the incidence of nosocomial COVID-19 with respect to the different types of health structures: hospitals providing acute and general care observe a lower incidence of care-related infectious cases compared to residential health care facilities and psychiatric therapeutic communities.

Certainly, compared to other types of HAIs, it is much more complex due to the variability of the incubation period, the possibility of transmission by asymptomatic person (which make any type of tracing difficult), and the great diffusion (especially in some pandemic periods) within the community [8].

Numerous studies [9,10,11] proposed strict prevention measures with indications regarding not only the use of PPE, the isolation of positive subjects and social distancing, and the hygiene and sterilization procedures, but also the correct distribution of hospital structures, adequate staff rostering, the use of negative pressure areas, entry and exit routes, and the use of elevators.

In 2021, indications were issued by the European Center for Disease Prevention and Control (ECDPC) in order to classify the cases that tested positive for SARS-CoV-2 during hospitalization [12].

In any case, the guidelines provided by the ECDPC appear generic, are essentially based on symptom onset (variable and subjective aspects), and do not consider further important elements (such as diagnostic tests or instrumental investigations).

As previously mentioned, nosocomial SARS-CoV-2 infection undoubtedly has wide repercussions in the medical-legal field. Primarily, in COVID-19 cases, we must understand whether the infection actually occurred in the hospital setting (nosocomial event) or if it is more likely a community transmission that has had clinical expression only after hospitalization.

It is essential to assess whether all measures to prevent infectious risk have been correctly applied and whether the infection can be considered “inevitable” (i.e., not due to a mistake by the healthcare staff) or “avoidable” (therefore related to an alleged health responsibility).

The correct framing of the case and compliance with all protection procedures have a fundamental defensive value in claims for professional liability due to the occurrence of HAIs.

At present, there is a risk of a large amount of dispute for cases of SARS-CoV-2 infection occurring in the nosocomial setting [13]. The most controversial aspect is the evaluation of patients who tested negative for the first swab (at the first hospital access), with subsequent positive tests during hospitalization. The possible negativity of the first hospital swab is not sufficient to define a case as unequivocally nosocomial. In fact, nasopharyngeal swabs represent operator-dependent sampling and can be falsely negative due to an incorrect execution procedure. According to the literature, [14] nasopharyngeal swabs require rigorous and careful sampling and conservation procedures. On the other hand, during the course of SARS-CoV-2 infection, it is possible that the amount of virus in the upper airways is minimal [15] (especially in the initial stages) and therefore not always detectable through commonly used diagnostic tests (in fact, RT-PCR requires at least 250 genomic copies/mL). Therefore, the analysis of COVID-19 cases diagnosed during hospitalization is a necessary prerequisite from a medical-legal point of view to avoid excessive overestimation of nosocomial cases with an undue increase in costs related to medicolegal disputes. The key point is to understand which of the COVID-19 cases diagnosed during hospitalization are truly nosocomial.

The objective of this study was to propose classification criteria to frame COVID-19 cases diagnosed during hospitalization (after initial negative screening) and analyze the cases of patients who died at one of the largest hospitals in northwestern Italy.

## 2. Materials and Methods

In order to standardize the methodology, the primary objective was to create a classification that could frame the COVID-19 cases diagnosed in the hospital (first negative entry swab, with subsequent positive test during hospitalization) into five categories: nosocomial, probably nosocomial, indeterminate, probably community, and certainly community.

Following bibliographic research, the classification proposed by the European Center for Disease Prevention and Control [12] was used as a reference.

The categorization, proposed in 2020, is essentially based on incubation times and has some “subjective” aspects (for example, the personal criterion of suspicion of community or nosocomial transmission–i.e., “strong suspicion of community/healthcare transmission”) that are difficult to standardize. It was therefore considered appropriate to implement and update the aforementioned classification, considering additional clinical and radiological aspects (radiological characteristics of COVID-19, as described by authoritative bibliographic sources [16]).

In the first instance, data from chest CT scans (any ground glass aspects, mono or bilateral inflammatory pulmonary thickening) were taken into consideration at hospital admission (within 24–36 h). Alternatively, if a CT was not available, chest radiographic findings (thickening, mono or bilateral opacities) were evaluated.

With regard to incubation times, several recently published studies were taken into consideration [17,18,19,20], taking into account the time frame in which the onset of symptoms occurs in the majority of cases (approximately 95%). In particular, the time limits referred to in the classification were essentially the fourth day (lower limit) and the fourteenth day (upper limit). Specifically, based on the meta-analyses cited [18,19,20,21], the 95% confidence interval was not less than four days. Therefore, in over 95% of cases, the onset of symptoms occurred after at least 4 days. In consideration of the medical-legal purpose (i.e., addressing the problem of litigation relating to presumed healthcare-related infections), considering a lower time limit (second or third day) would have led to an underestimation of community cases and an excessive overestimation of nosocomial cases.

On the other hand, the fourteenth day was considered as the upper limit (contemplating the time margin within which the infection almost certainly occurs from the moment of contagion), as also suggested by the ECDPC [12].

In order to define the classification criteria, a multidisciplinary team was set up which included specialists in Forensic Medicine, Infectious Diseases, and Biostatistics. A first draft was initially proposed and was reviewed by a Medical-Legal Reviewer and Infectious Disease Consultant on the basis of the published scientific evidence [12]. Subsequently, the medical-legal classification was drawn up, defined as follows:

**CASES RELATED TO ASSISTANCE**

- Positivity 14 days from entry, absence of infectious/respiratory symptoms at entrance, absence of pathological radiological findings at entrance.

**CASES PROBABLY RELATED TO ASSISTANCE**

- Positivity between 4 and 14 days from entrance, absence of infectious/respiratory symptoms at entrance, absence of pathological radiological findings at entrance.

**INDETERMINATE**

- Positivity after 14 days with infectious/respiratory symptoms at entrance; infectious/respiratory symptoms and radiological pathological findings at entrance; or radiological pathological findings without symptoms at entrance.

**PROBABLY COMMUNITY CASES**

- Positivity within 4 days without infectious/respiratory symptoms at entrance and without pathological radiological findings at entrance.
- Positivity between 4 and 14 days from entrance with infectious/respiratory symptoms at entrance; infectious/respiratory symptoms and radiological pathological findings at entrance; or radiological pathological findings without symptoms at the entrance.

**COMMUNITY CASES**

- Positivity within 4 days of nosocomial entry with infectious/respiratory symptoms at entry; infectious/respiratory symptoms and radiological pathological findings at entrance; or radiological pathological findings without symptoms at entrance.

Subsequently, all the deaths with SARS-CoV-2 infections identified during hospitalization (after initial negative screening) in a large university hospital in Northwest Italy were taken into consideration in the period from the beginning of the pandemic (February 2020) until 31 December 2021.

With regard to the definition of the criteria for the inclusion of the cohort, it was decided to include all deaths that occurred in the period of interest. Several groups were selected and are listed as follows: deaths with first positive SARS-CoV-2 swab, deaths with initial negative SARS-CoV-2 swab and subsequent positive tests during hospitalization. In addition, the total number of deaths (from any cause of death) in the period under study was considered.

The data collected included the following: age and sex of deceased subjects, date of hospital admission, date of death, hospital certification of cause of death, date and results of all nasopharyngeal swabs performed at the hospital, anamnestic data (near and remote pathological), reason for admission and symptoms at hospital admission, radiological examinations at admission and during hospitalization, description of the in-hospital clinical course. With regard to nasopharyngeal swabs, only tests performed by molecular research (RT-PCR) were considered.

Essentially four different time bands were considered: from 20 February 2020 to 17 May 2020 (Period 1); from 18 May 2020 to 19 November 2020 (Period 2); from 20 November 2020 to 15 March 2021 (Period 3) and from 16 March 2021 to 31 March 2021 (Period 4).

Statistical analysis: The discrete variables were presented as absolute numbers and percentages, while the continuous variables were reported as mean with standard deviation and as median with range. To compare the four periods, the Chi-square test or Fisher’s test were used for the categorical variables and ANOVA was performed for continuous variables, using Bonferroni correction for comparisons of the single periods. The *p*-values < 0.05 were considered statistically significant and Stata software version 16.0 (Stata Corporation, College Station, TX, USA) was used for the analysis.

## 3. Results

### 3.1. Population under Study

A total of 4672 deaths occurred from 20 February 2020 to 31 December 2021, of which 1266 died with COVID-19 and 3789 patients entered with a negative swab and then died from any cause of death (Table 1).

There were 383 cases diagnosed in the hospital after negative entry tests, of which radiological data were available in 375 cases (98) and the symptoms described in 100% of cases.

In the period from 20 February 2020 to 17 May 2020, the total number of deaths was 792, of which 304 were COVID-19 cases; among these, 82 cases concerned patients with positive SARS-CoV-2 test during hospitalization after an initial negative test at hospital admission.

In the period between 18 May 2020 and 19 November 2020, the total deaths were 1148, with an estimated 247 cases of COVID-19 death and, among these, 45 cases diagnosed during hospitalization (after initial negative test).

From 20 November 2020 to 15 March 2021 (second wave) the deaths at the hospital were 977, of which there were 510 COVID-19 and 214 cases negative for a first nasopharyngeal swab and positive for a subsequent molecular test in-hospital.

Finally, in the period from 16 March 2021 to 31 December 2021, a total of 1755 deaths were reported, of which 205 COVID-19 cases and, among these, 42 cases concerned SARS-CoV-2 cases diagnosed during hospitalization, after an initial negative test.

### 3.2. Overall Analysis from 20 February 2020 to 31 December 2021

Overall, 383 patients with a first negative RT-PCR nasopharyngeal swab at hospital admission died with a positive SARS-CoV-2 test found during hospitalization.

Overall, the average age was 81.83 (standard deviation–SD 9.04) with a percentage of men of 57% and a percentage of women of 43%.

The mean number of negative nasopharyngeal swabs before the hit was 2.83 (SD 1.91); the mean hospital stay before the positive test was 12.86 days (SD 9.90) with a median of 10 days (minimum 0 days, maximum 73 days).

Based on the classification criteria, only 41 cases (11%) were to be considered nosocomial, while 71 cases (19%) were classified as probably nosocomial, 69 cases (18%) as indeterminate, 166 cases (43%) were classified as probably community, and 36 cases (9%) as certainly community.

Deaths with nosocomial infection constituted the following: 3.23% (41/1266) with respect to the total number of COVID-19 deaths, 1.1% (41/3789) with respect to those who entered the hospital with a negative swab, and 0.87% (41/4672) with respect to the total of deaths from any cause of death.

Taking into consideration also the probably nosocomial cases, the percentage (nosocomial cases plus the probably nosocomial cases) is equal to 8.8% compared to deaths with COVID-19 (*n* = 1266) and 2.39% compared to the total number of deaths from any cause of death. Taking into account the patients who entered the hospital with the first negative swab, the percentage of nosocomial and probably nosocomial cases is 2.95%.

### 3.3. Analysis from 20 February 2020 to 17 May 2020

The time frame that elapses from the beginning of the spread of SARS-CoV-2 to 17 May 17 2020 roughly corresponds to the so-called first pandemic wave, which occurred in Italy.

The total number of deaths was 792 and the COVID-19 patients who died were 304, while the patients that entered with a negative swab and then died from any cause of death were 570.

During this period, 82 deaths (21%) were found among patients with a SARS-CoV-2 positive test during hospitalization, after an initial negative finding.

During this period, the average age of the subjects was 79.99 years (SD 11.17). In this group, the number of men was 50 (61%).

The mean of negative nasopharyngeal swabs prior to the hit was 1.52 (SD 0.76). The mean hospital stay before the positive test was 14.16 days (SD 12.73) with a median of 11 days.

**Figure 1:**
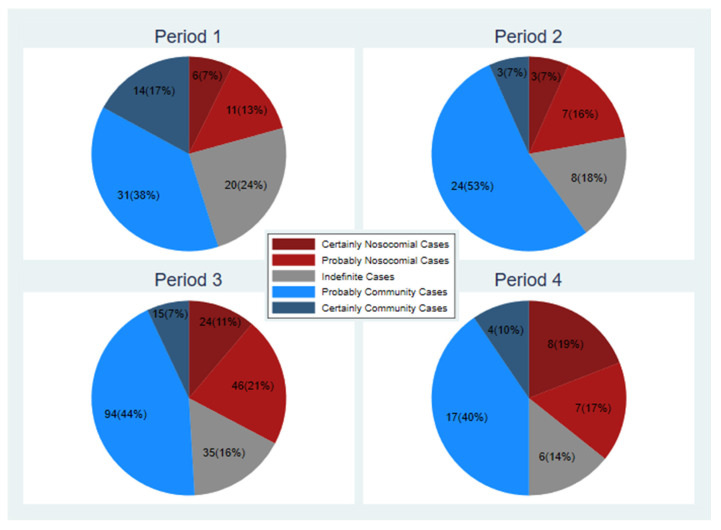
Representation of the cases divided into five classes and divided into different phases of analysis.

According to the classification criteria, six cases out of 82 (7%) were defined as HAIs. In addition, 11 cases (14%) were probably nosocomial cases, 20 cases (i.e., 24%) were indeterminate, 31 cases (38%) were probably community and 14 (17%) were classified as certainly community (Figure 1, period 1).

Nosocomial-infected deaths constituted 1.97% (6/304) of the overall number of deaths with SARS-CoV-2 infection, 0.75% (6/792) of total deaths from any cause of death and 1.05% (6/570) compared to those who entered the hospital with a negative swab.

Taking into consideration also the probably nosocomial cases, the percentage (relative to nosocomial cases plus probably nosocomial) is equal to 5.59% compared to the total of deaths in all COVID-19 subjects (i.e., 304 cases) and 2.14% compared to the total number of deaths (792) from any cause of death. Taking into account the patients who entered the hospital with the first negative swab, the percentage of nosocomial and probably nosocomial cases is 2.98%.

### 3.4. Analysis from 18 May 2020 to 19 November 2020

The period from 18 May to 19 November 2020, in Italy, is concise with the phase of net reduction in viral circulation (after the lockdown period) and the subsequent start of the second wave.

The total number of deaths was 1148, the COVID-19 positive deaths were 247, while the patients who entered with a negative swab and then died from any cause of death were 946.

During this period, there was a sharp decrease in deaths of patients negative for a first nasopharyngeal swab and positive for a subsequent molecular test in-hospital: 45 cases were found (i.e., 12% compared to the total of 383).

The average age in this group of patients was 83.62 (SD 6.91), with a percentage of male subjects equal to 60% (27 subjects).

The mean number of negative swabs prior to the positive test was 2.67 (SD 1.21); the mean time to positive swab was 10.64 days (SD 6.91) with a median of 8 days (minimum 1 day, maximum 33 days).

A total of three cases (7% out of 45) were classified as HAIs and 7 (15%) were classified as probably nosocomial cases. A further eight cases (18%) were indeterminate. On the other hand, 24 patient cases (53%) were thought to be probably community and three cases (7%) were certainly of community relevance (previous infection before admission) (Figure 1).

Nosocomial-infected deaths constituted 1.21% (3/247) of the overall number of deaths with SARS-CoV-2 infection, 0.26% (3/1148) of total deaths from any cause of death, and 0.31% (3/946) compared to the subjects who entered the hospital with a negative swab.

Taking into consideration also the probably nosocomial cases, the percentage (relative to nosocomial cases plus probably nosocomial) is equal to 4.04% compared to the total of deaths in all COVID-19 subjects (i.e., 247 cases), and 0.87% of the total number of deaths from any cause of death. Taking into account the subjects who entered the hospital with the first negative swab, the percentage of nosocomial and probably nosocomial cases is 1.05%.

### 3.5. Analysis from 20 November 2020 to 15 March 2021

The period between 20 November 2020 and 15 March 2021 is the second wave of the pandemic in Italy with a significant increase in the spread of the virus and a marked overload of the hospital structures. Essentially, so far, this temporal juncture can be considered the worst pandemic phase that has occurred in Italy.

The total number of deaths was 977, the COVID-19 positive deaths were 570, while the patients who entered with a negative swab and then died from any cause of death were 681.

During this period, a high number of deaths of patients with a positive test after negative entry tests were recorded during hospitalization: 214 cases were detected (equal to 56% of the total of 383).

The average age of this group of patients was 81.57 (SD 8.80) with a percentage of the male population equal to 55% (118 patients). The mean number of negative swabs before positive swab was 3.11 (SD 1.76). The mean number of hospital days before viral detection was 12.48 (SD 8.67) with a median of 10.5 days (min 2 days, maximum 67 days).

According to the proposed classification, 24 cases (11%) were nosocomial and 46 cases (22%) were classified as probably nosocomial; furthermore, 35 cases (16%) were classified as indeterminate. On the other hand, 94 cases (44%) were probably community and 15 cases (7%) were community cases (Figure 1, period 3).

Deaths with certainly nosocomial infection constituted 4.7% (24/510) of the total number of deaths with SARS-CoV-2 infection, 2.45% (24/977) of the total deaths from any cause of death, and 3.52% (24/681) compared to those who entered the hospital with a negative swab.

Taking into consideration also the probably nosocomial cases, the percentage (relative to nosocomial cases plus probably nosocomial) was equal to 13.72% compared to the total of deaths in all COVID-19 subjects (i.e., 510 cases) and 7.16% compared to the total number of deaths from any cause of death. Taking into account the patients who entered the hospital with the first negative swab, the percentage of nosocomial and probably nosocomial cases was 10.28%.

### 3.6. Analysis from 16 March 2021 to 31 December 2021

The period from 16 March 2021 to 31 December 2021 represented a phase of viral defervescence in Italy, with a decrease in cases and a reduction in the clinical severity of the disease. In fact, an effective large-scale vaccination campaign was advocated during this period.

The total number of deaths was 1755, the COVID-19 positive deaths were 205, while the patients who entered with a negative swab and then died from any cause of death were 1592.

In this temporal context, 42 deaths of patients (11% out of the total of 383) with positive test were recorded during hospitalization (after negative entry tests).

The average age in this group of patients was 84.83 years (SD 6.47) with a percentage of male subjects equal to 60% (25 cases).

The mean number of negative swabs before positive swab was 4.10 (SD 3.12); the mean time before in-hospital diagnosis (following an initial negative result) was 14.67 days (SD 11.76) with a median of 10 days (min 1 days, maximum 53 days).

Under the proposed classification, 8 cases (19%) were classified as nosocomial cases and 7 patients (17%) were classified as probably nosocomial cases. Six cases (14%) were indeterminate cases. Instead, 17 cases (40%) were probably community and four cases (10%) appeared of community origin (Figure 1, period 4).

Deaths with nosocomial infection constituted 3.90% (8/205) of the total number of deaths with SARS-CoV-2 infection, 0.45% (8/1755) of the total deaths from any cause of death and 0.50% (8/1592) of the subjects who entered the hospital with a negative swab.

Taking into consideration also the probably nosocomial cases, the percentage (relative to nosocomial cases plus probably nosocomial) was equal to 7.31% compared to the total of deaths in all COVID-19 subjects (i.e., 205 cases) and 0.85% compared to the total number of deaths from any cause of death. Taking into account the subjects who entered the hospital with the first negative swab, the percentage of nosocomial and probably nosocomial cases was 0.94%.

When the four waves were compared, we found statistically significant differences in terms of age (*p* = 0.0184), observing that patients at wave 4 were older compared to those at first wave. Interestingly the mean number of negative swabs before becoming positive increased over the waves (*p* < 0.001). Concerning symptoms and radiology, we observed statistically significant differences among waves in terms of presence of infectious symptoms (*p* = 0.019, wave 1: 44%, wave 4: 29%) as well as in terms of pathological radiology (*p* < 0.001 wave 1: 62%, wave 4: 50%).

## 4. Discussion

The problem related to the damage resulting from nosocomial infections, more correctly defined as healthcare-related infections, has entered the pandemic context. In cases of positive SARS-CoV-2 test during hospitalization, the Healthcare Facility may be called to respond for professional liability, unless it proves that the infection was not contracted in the nosocomial setting (falling within the category of community cases with initial false negative diagnostic test) or to have slavishly observed all the HAIs prevention protocols [21].

The first part of this study has the purpose of proposing a standardized tool that can help to correctly frame the case and understand whether the infection is of nosocomial or community relevance.

The second phase of the study collected data relating to the deaths of patients with a positive test during hospitalization (after negative entry tests), in a large university hospital in Northwest Italy; after skimming the documents, the medical-legal evaluation classification was applied.

The results of this research project show that the infection in a hospital setting (after initial negative nasopharyngeal swab) mainly concerned male patients (57%); these data appear to be consistent with the data already published in the literature [2,22,23]. The average age was 81.83 years, higher than that described by other scientific studies [2,22,23]. In addition, the average time between hospital entry and the positive nasopharyngeal swab (which roughly also corresponds to the evident clinical manifestation of the disease) was 12.86 days, with a median of 10 days. A scientific study [22] reports an average time between hospitalization and symptomatic onset of 24.5 days.

Based on the statistical analysis, a total of 383 cases of COVID-19 positive test during hospitalization (after negative entry tests) were tracked from 20 February 2020 to 31 December 2021, after an initial negative screening. They represent 8.2% of total deaths, and 10.1% of cases entering the hospital with a negative SARS-CoV-2 swab.

Of these, during the entire period under analysis, only 41 cases were classified as healthcare-related infections (11% compared to 383 deaths), and instead 71 cases (19%) were probably considered nosocomial.

However, this study shows that only a small number of COVID-19 cases diagnosed during hospitalization have a purely nosocomial origin.

From a medical-legal point of view, these data appear extremely interesting: it demonstrates, in fact, that a diagnosis of COVID-19 during hospitalization (even in the face of initial negative screening) cannot and must not be considered, a priori, a care-related infection. These cases require an in-depth clinical evaluation that takes into account the time interval between hospital admission and diagnosis, the symptoms and radiological findings (where available), and further specific and individual data (possible tracing, further cases in the specific department, etc.).

Based on the analysis performed, considering the period from 20 February 2020 to 31 December 2021, the percentage of nosocomial cases compared to the total of deaths from any cause of death is minimal, i.e., 0.87%. On the other hand, if we consider the patients who entered the hospital with a negative swab, the percentage of nosocomial cases is 1.08%.

Also taking into account the probably nosocomial cases, the percentage is 2.39% of the total number of deaths from any cause of death. On the other hand, with regard to patients who entered the hospital with the first negative swab, the percentage of nosocomial cases plus the probably nosocomial cases is 2.95%.

These values are in line with the series published in the literature. For example, a recent review [24] that considered 5920 articles published in the literature reported a high proportion of nosocomial COVID-19 with a percentage between 18.5 and 65%, before the implementation of measures of prevention (therefore during the onset of the pandemic emergency). According to this review, the majority of studies conducted after the implementation of infectious containment protocols report a low percentage (less than 6%) and only a few reports describe higher percentages between 15.40 and 53.60%.

In addition, based on a review by Ndongu et al. [24], the percent range of nosocomial SARS-CoV-2 was 6.48–35.00%, 0–46.29%, 4.60–19.00%, and 0.02–53.60% in Spain, the United Kingdom, Canada, and the United States, respectively.

For example, Landoas et al. [22] reported that during the first wave of the pandemic, the rate of nosocomial COVID-19 was 5.4% in a French university hospital. A statistical model conducted by Public Health England (PHE) predicted that about 20% of hospitalized COVID-19 cases during the first wave are nosocomial [25].

A study [26] described a rate of health care-related SARS-CoV-2 infections of 18.5%. A study conducted in three Scottish hospitals reported an 11% rate of healthcare-related infections [2]. Furthermore, an analysis conducted in UK and Italian hospitals (during the first pandemic wave) found that the percentage of hospital-acquired COVID-19 cases was 12.5%. In addition, a British study, conducted from March to December 2020, found a percentage of healthcare-related infections equal to 0.5% (0.34–0.74) in patients admitted to “acute National Health Service trust” [8]. Finally, a recent Spanish study, published in 2022, analyzed the onset of SARS-CoV-2 infections related to care in the San Vincent del Raspeig Hospital in Alicante from 21 January to 15 March 2022, and found that 60.2% of patients admitted to the hospital were diagnosed with nosocomial COVID-19 [27].

In any case, a full comparison between the analysis of this discussion and the aforementioned studies published in the literature is not possible: first of all, because the sample selected by us only concerns deceased patients; moreover, the current purposes concern medical-legal aspects. On the other hand, all the studies published in the literature essentially deal with clinical and epidemiological aspects with a sample that also concerned patients who were subsequently cured and not just deceased persons.

However, we must consider that all the studies published in the literature (and also the present treatment) may be burdened with an imperfect estimate of the nosocomial incidence of SARS-CoV-2 infection: Due to the clinical heterogeneity of the infection, the use of narrow cut-offs from the time of hospital admission can lead to an overestimation of community cases; instead, the possible entry into the healthcare facility during a completely asymptomatic phase can lead to an overestimation of nosocomial cases [8].

This study also shows a tangible difference in the spread of COVID-19 related to assistance based on the different time periods.

According to the bulletins of the Istituto Superiore di Sanità [28], the greatest viral spread, pandemic severity and health management difficulties were recorded in Italy during the first pandemic wave (February–May 2020) and the second wave (autumn 2021). Subsequently, the vaccination campaign led to a marked improvement in terms of avoided cases and hospitalization [29].

During the first pandemic wave (which took place in Italy until about mid-May 2020), the hospitals had to face an exceptional and unexpected emergency were insufficient. In this context, there are spaces for isolation, equipment, supplies, and individual safety devices. In situations of absolute emergency, even the utmost diligence cannot be considered sufficient to avoid infections, due to the insurmountable management difficulties [1]. Due to the difficulties in prevention, it is reasonable to expect an increase in cases of nosocomial spread of COVID-19.

According to this study, a higher percentage of positive SARS-CoV-2 test was recorded during hospitalization (after initial negative screening) from 20 February 2020 to 17 May 2020, however the percentage of nosocomial and probably nosocomial (and also the ratio between these cases and the total deaths) is not tangibly higher than the average values of the whole period under study. However, if we consider the periods of lower viral circulation (May–October 2020 and March–December 2021), the percentage of the first pandemic phase is higher. In terms of medicolegal disputes, the certainly nosocomial cases could be “justified” by the particular and unexpected health emergency that made extremely complicated and sometimes impracticable a suitable strategy for containing the infectious risk from COVID-19.

According to this study, the higher incidence of nosocomial and probably nosocomial cases coincides with the second wave of the pandemic (late 2020 and early 2021). Certainly during this period the health personnel had the time, the tools, and even the scientific knowledge to cope with the pandemic emergency. It is also true that during this pandemic phase there has been an enormous viral spread with a notable increase in hospital admissions with rapid hospital overload.

During this period, protocols relating to the containment of the risk of contagion were already widespread and the health structures had now been provided with tools and means to deal with the pandemic. Any hospital overload and the material impossibility to carry out adequate patient isolation (in consideration of the marked increase in hospitalization and the necessary duty of care), if proven, can lighten the hospital’s responsibility. However, will this aspect be sufficient to consider the care-related infection not otherwise avoidable? Unfortunately, this question cannot have a suitable and universal answer for all episodes. Each case must be carefully evaluated by a team that includes the Health Department, the medical-legal and also specialists in infectious diseases, analyzing every aspect related to the hospitalization and the hospital environment.

Lastly, the periods from 18 May 2020 to 19 November 2020 and from 16 March 2021 to 31 December 2021 were characterized by a low incidence of COVID-19 cases diagnosed during hospitalization (after negative screening), and a minimal percentage of nosocomial and probably nosocomial cases (compared to the total deaths and deaths of patients hospitalized with first negative nasopharyngeal swab). The first temporal moment (spring-autumn 2020) corresponds to the phase of lower circulation and viral spread, when hospital admissions were limited and there had already been an adequate supply of tools and measures to cope with the pandemic emergency. This condition explains the reduced rate of HAIs. On the other hand, since March 2021, although the viral spread was still significant, hospitalization and the severity of COVID-19 cases have decreased due to the effectiveness of the treatments and vaccination. These aspects may justify the marked reduction in healthcare-related SARS-CoV-2 deaths starting in the spring of 2021.

In these temporal contexts, although the cases related to assistance are limited, it is reasonable to state that the possible disputes (COVID-19 nosocomial certain or probable) are difficult to “justify” and could be traced to the responsibility of the hospital. In fact, during these periods, beyond specific and particular cases, it is difficult to trace any objective and insurmountable problems (such as scarce resources, overcrowding, and scarce initial scientific knowledge) that may justify nosocomial infections.

Overall, the study provides an updated classification of COVID-19 cases diagnosed during hospitalization to establish any nosocomial or community origin. The study shows that from 20 February2020 to 31 December 2021, a concrete number of deceased patients became positive during hospitalization, but only a small percentage of these patients were considered nosocomial or probably nosocomial cases.

However, this study had some limitations. First, the sample considered a population of deceased patients. Second, it was not possible to perform a genomic evaluation to identify the variant of SARS-CoV-2 responsible for infection in each individual case. Furthermore, the classification was unsatisfactory in all the cases. For example, the indeterminate group does not allow the proposal of a valid and comprehensive preliminary assessment. The classification criteria represent a useful preliminary evaluation tool, but we believe that a specific in-depth evaluation is necessary to analyze the peculiarities of each individual case.

## 5. Conclusions

Healthcare-related SARS-CoV-2 infection is an issue of particular concern during the pandemic. The same has important repercussions on the National Health System, also representing a source of medical-legal disputes.

In this study, we propose classification criteria to frame COVID-19 cases diagnosed during hospitalization (after initial negative screening) and analyze the case history of deceased patients with COVID-19 diagnosed during hospitalization (after initial negative SARS-CoV-2 screening) in a large university hospital in Northwest Italy. It has been shown that, from 20 February 2020 to 31 December 2021, a concrete number of deceased patients were diagnosed with COVID-19 during hospitalization, but only a small percentage of these can be considered nosocomial or probably nosocomial.

The highest incidence of SARS-CoV-2 infections, or probably HAIs, was recorded during the first pandemic wave and, above all, during the second wave (i.e., in the period of autumn/winter 2020–2021) when hospitals in Italy were burdened by a clear overcrowding due to the severe viral spread.

## Figures and Tables

**Table 1:** Descriptive statistical representation of the results obtained in the various pandemic periods.

	20 February 2020–17 May 2020	18 May 2020–19 November 2020	20 November 2020–15 March 2021	16 March 2021–31 December 2021	Overall	
Total deaths	792	1148	977	1755	4672	
COVID-19 deaths	304	247	510	205	1266	
Positive COVID-19 test during hospitalization	82	45	214	42	383	*p*-value
Positive COVID-19 test during hospitalization, Number (%)	82 (21%)	45 (12%)	214 (56%)	42 (11%)	383 (100%)	
Classification, Number (%)						
Nosocomial cases	6 (7%)	3 (7%)	24 (11%)	8 (19%)	41 (11%)	0.212
Probably nosocomial cases	11 (14%)	7 (15%)	46 (22%)	7 (17%)	71 (19%)	0.387
Indeterminate	20 (24%)	8 (18%)	35 (16%)	6 (14%)	69 (18%)	0.384
Probably community cases	31 (38%)	24 (53%)	94 (44%)	17 (40%)	166 (43%)	0.388
Community cases	14 (17%)	3 (7%)	15 (7%)	4 (10%)	36 (9%)	0.077
Age, Mean (SD))	79.99 (11.17) ^d^	83.62 (6.91)	81.57 (8.80)	84.83 (6.47) ^a^	81.83 (9.04)	0.0184
Male, Number (%)	50 (61%)	27 (60%)	118 (55%)	25 (60%)	220 (57%)	0.782
Number of negative swabs before of the first positive, average (DS)	1.52 (0.76) ^b,c,d^	2.67 (1.21) ^a,d^	3.11 (1.76) ^a,d^	4.10 (3.12) ^a,b,c^	2.83 (1.91)	<0.001
Time to positivity, Mean (DS)	14.16 (12.73)	10.64 (6.91)	12.48 (8.67)	14.67 (11.76)	12.86 (9.90)	0.1460
Median (Min-Max)	11 (0;73)	8 (1;33)	10.5 (2;67)	10 (1;53)	10 (0;73)	

^a^: Statistically significant comparison compared to wave 1 based on post-hoc analysis with Bonferroni correction. ^b^: Statistically significant comparison compared to wave 2 based on post-hoc analysis with Bonferroni correction. ^c^: Statistically significant comparison compared to wave 3 based on post-hoc analysis with Bonferroni correction. ^d^: Statistically significant comparison compared to wave 4 based on post-hoc analysis with Bonferroni correction. The *p*-values refer to ANOVA for continuous variables and to Chi-square test of Fisher’s exact test for categorical variables.

## Data Availability

Not applicable. Data were extrapolated from hospital databases.
